# Transcriptomic Analysis of the Effects of Hydroxysafflor Yellow A on hUC-MSC Senescence via the ECM–Receptor Interaction Pathway

**DOI:** 10.3390/ijms26199579

**Published:** 2025-10-01

**Authors:** Siyun Wang, Qi Zhu, Xueer Feng, Xinghua Chou, Tao Lu

**Affiliations:** School of Life Science, Beijing University of Chinese Medicine, Beijing 102488, China

**Keywords:** hydroxysafflor yellow A, hUC-MSCs, senescence

## Abstract

This study investigated the mechanism of hydroxysafflor yellow A (HSYA) on senescent human umbilical cord mesenchymal stem cells (hUC-MSCs) through transcriptome sequencing. HSYA treatment identified 2377 differentially expressed genes (DEGs). Gene Ontology (GO) and Kyoto Encyclopedia of Genes and Genomes (KEGG) analyses revealed that these DEGs were primarily enriched in cell adhesion regulation and the extracellular matrix (ECM)–receptor interaction pathway. Gene Set Enrichment Analysis (GSEA) and protein–protein interaction (PPI) network analysis corroborated the central role of ECM–receptor interaction signaling, and Key Driver Analysis (KDA) revealed 10 core regulatory genes (e.g., ID1, SMAD3, TGFB3). SA-β-gal staining showed that HSYA significantly reduced senescence-associated β-galactosidase activity. Flow cytometry showed no significant changes in cell cycle distribution. Western blot analysis indicated that HSYA treatment reduced the protein expression level of p16 without significantly altering p53 levels. Furthermore, HSYA significantly attenuated intracellular reactive oxygen species (ROS) accumulation. qPCR validation demonstrated that HSYA significantly upregulated ID1, GDF5, SMAD3, and TGFB3 while downregulating BMP4, TGFB2, and CCN2. These findings indicate that HSYA modulates genes associated with the ECM–receptor interaction pathway, potentially contributing to improved ECM homeostasis in senescent hUC-MSCs.

## 1. Introduction

Human umbilical cord mesenchymal stem cells (hUC-MSCs) are multipotent stem cells capable of self-renewal and differentiation [1]. While their non-invasive harvesting and low immunogenicity confer clinical advantages [2,3], and they are widely utilized in treating organ injury, neurological, and endocrine diseases [4,5,6], replicative senescence during in vitro expansion significantly impairs proliferation, differentiation, and immunomodulatory functions [7,8], thereby reducing therapeutic efficacy and increasing safety risks [9]. Critically, the extracellular matrix (ECM) provides essential physical and biochemical cues that regulate stem cell fate and function. Alterations in the ECM composition and disrupted signaling through ECM–receptor interactions are critically involved in driving cellular senescence programs [10,11,12]. Senescent MSCs exhibit dysregulated ECM production and remodeling, which in turn can further exacerbate senescence and impair their functional capacities [13,14].

In the context of anti-aging strategies, approaches such as gene editing technologies, pharmacological interventions, and exosome-based therapies not only enhance the efficiency and quality of hUC-MSCs during in vitro expansion but also improve their post-transplantation survival rate, engraftment capacity, and therapeutic efficacy [15,16,17,18,19]. Of these approaches, pharmacological interventions using small-molecule compounds have emerged as a promising and versatile strategy to combat MSC senescence, with mechanisms spanning antioxidant defense, epigenetic regulation, metabolic reprogramming, and senolysis [20,21,22].

Hydroxysafflor yellow A (HSYA), a bioactive chalcone derivative isolated from the traditional Chinese herb *Carthamus tinctorius* L., has demonstrated significant antioxidant and anti-inflammatory properties through multiple pharmacological mechanisms [23]. Accumulating evidence indicates that HSYA exhibits protective effects across various pathological conditions. In cerebrovascular disorders, HSYA administration attenuates cerebral ischemia–reperfusion injury by suppressing the NF-κB signaling cascade and has been successfully translated into clinical practice as an injectable formulation for ischemic stroke management [24]. Regarding anti-fibrotic activity, HSYA effectively mitigates organ fibrosis progression through downregulation of the TGF-β/Smad pathway [25]. Furthermore, molecular studies have revealed that HSYA confers hepatoprotection via activation of the Nrf2/HO-1 antioxidant pathway [26], while its anti-inflammatory efficacy has been mechanistically linked to modulation of NLRP3 inflammasome activity [27]. These findings suggest that HSYA may counteract cellular senescence through multiple pathways, potentially involving the regulation of oxidative stress and cell cycle arrest.

However, the precise molecular mechanisms underlying HSYA’s anti-senescence effects in hUC-MSCs, particularly whether and how it modulates the ECM–receptor interaction pathway, have not been fully delineated. Moreover, the impact of HSYA on the transcriptome and gene regulatory networks in senescent hUC-MSCs, with a focus on ECM-related processes, remains unclear.

In this study, we employed RNA sequencing combined with bioinformatic analysis to investigate the effects of HSYA on the global gene expression profile of senescent hUC-MSCs. Through differential gene expression analysis, Gene Ontology (GO) functional enrichment, and Kyoto Encyclopedia of Genes and Genomes (KEGG)pathway analysis, we elucidated the key signaling pathways and molecular network mechanisms through which HSYA alleviates senescence in hUC-MSCs. Notably, while previous investigations have primarily focused on the antioxidant or anti-fibrotic roles of HSYA, this study explored its anti-senescence effects in hUC-MSCs via transcriptomic sequencing, with results particularly highlighting the potential critical involvement of the ECM–receptor interaction pathway. Our findings suggest that HSYA exerts its regulatory effects in delaying MSC senescence through a p53 protein level-independent regulatory mechanism, providing novel insights for combating MSC senescence and advancing regenerative medicine strategies.

## 2. Results

### 2.1. Differential Gene Expression Analysis

**Figure 1:**
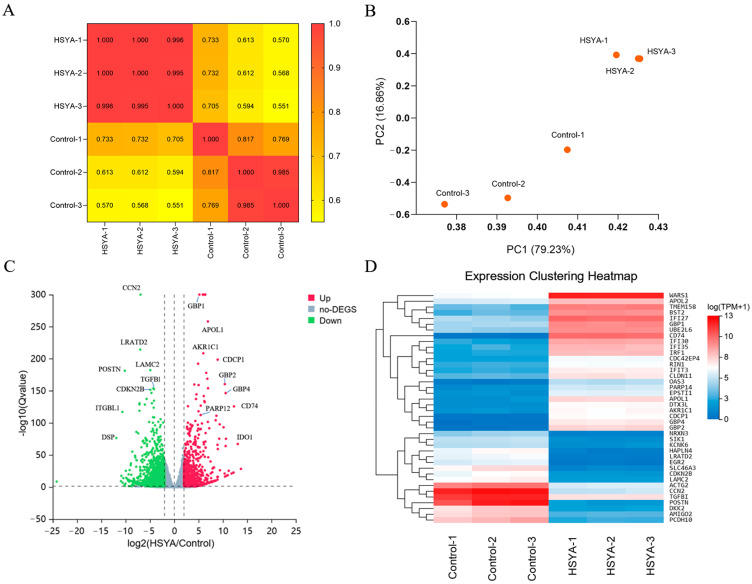
Differential Gene Expression Analysis. (**A**) Correlation heatmap of RNA-seq data across different sample groups. (**B**) PCA analysis of RNA-seq data from individual samples. (**C**) Volcano plot of DEGs. (**D**) Hierarchical clustering heatmap of the top 40 most significant DEGs.

The inter-sample gene expression correlation heatmap (Figure 1A) showed that samples within the HSYA group exhibited high correlation (r > 0.95), with tightly clustered data points, indicating excellent experimental reproducibility. In contrast, the Control group displayed a lower correlation coefficient (r > 0.75) and greater dispersion, possibly due to the inherent heterogeneity of senescent hUC-MSCs, leading to some variability in gene expression among replicates.

Principal component analysis (PCA, Figure 1B) further supported these findings, revealing distinct gene expression profiles between the Control and HSYA groups. Additionally, the low correlation between samples from different treatment groups aligned with the PCA results, reinforcing the observed inter-group differences.

In this study, we identified a total of 2377 differentially expressed genes (DEGs), including 878 upregulated genes and 1499 downregulated genes, using the screening criteria of *p* < 0.05 and |log_2_FC| ≥ 1.5. Figure 1C displays the volcano plot of DEGs, where red dots represent significantly upregulated genes, blue dots indicate significantly downregulated genes, and gray dots denote genes with no significant differences. The expression heatmap of the top 40 most significant DEGs (Figure 1D), with colors ranging from blue (low expression) to red (high expression). The samples clustered into two distinct groups, reflecting expression differences under different conditions. Among these DEGs, genes such as WARS1, DTX3L, GBP1, APOL1, and AKR1C1 were upregulated, while CCN2, LRATD2, LAMC2, POSTN, and TGFB1 exhibited a downregulated trend.

### 2.2. GO and KEGG Analysis

**Figure 2:**
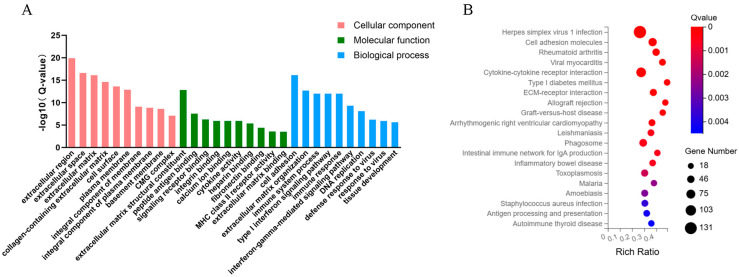
GO and KEGG Analysis. (**A**) The top 10 most significantly enriched GO terms in biological process (BP), molecular function (MF), and cellular component (CC) categories, ranked by Q-value. (**B**) Significantly enriched KEGG pathways.

Functional enrichment analysis of the 2377 DEGs identified between Control and HSYA groups revealed the top 10 significantly enriched terms in each GO category: Biological Process (BP), Molecular Function (MF), and Cellular Component (CC). In the BP category, cell adhesion was the most significantly enriched term, while ECMstructural constituent and extracellular region represented the predominant terms in MF and CC categories, respectively (Figure 2A). Functional analysis revealed that the DEGs are primarily associated with cell-microenvironment interactions, particularly regulating cell adhesion and mediating the structural organization and composition of ECMcomponents. The significant enrichment of these functionally related terms suggests that HSYA treatment may primarily affect cellular communication and ECM remodeling processes in senescent hUC-MSCs.

KEGG pathway enrichment analysis (Figure 2B) revealed significant enrichment of DEGs in 334 pathways, with 20 pathways meeting the significance threshold. Notably, the effects of HSYA were found to be primarily enriched in the ECM–receptor interaction pathway.

### 2.3. GSEA

**Figure 3:**
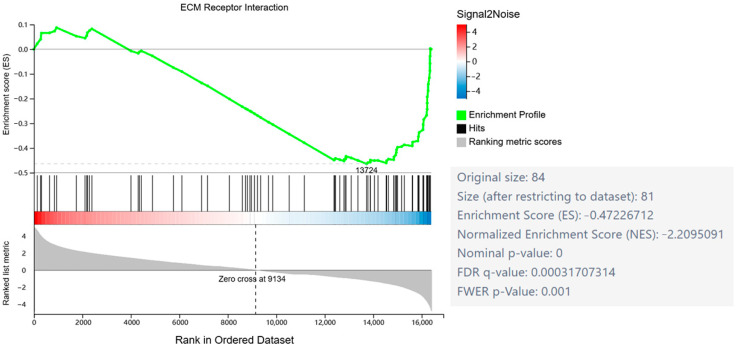
Gene Set Enrichment Analysis (GSEA). GSEA enrichment plot of the ECM–receptor interaction pathway.

Gene Set Enrichment Analysis (GSEA) is a computational method for gene expression analysis that evaluates the distribution patterns of predefined gene sets rather than individual genes. This approach enables detection of subtle but coordinated biological signals, where even genes with non-significant individual changes may collectively exhibit important biological relevance when analyzed as a functional set. As a complement to conventional differential expression analysis, GSEA provides enhanced capability to identify pathway-level perturbations that might otherwise be overlooked through single-gene assessment. GSEA revealed significant enrichment of the ECM–receptor interaction gene set in HSYA-treated samples (Figure 3), indicating that HSYA may regulate ECM homeostasis through modulation of this pathway.

### 2.4. PPI Network and KDA

**Figure 4:**
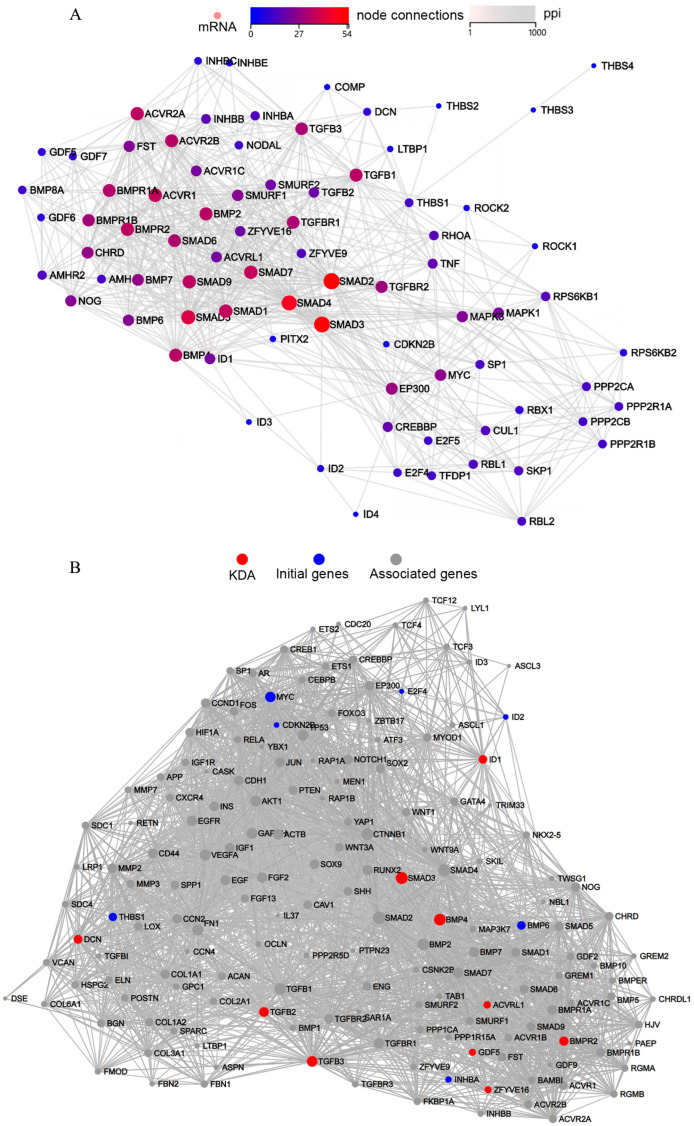
Protein–protein interaction (PPI) network and key driver analysis (KDA) of the ECM–receptor interaction pathway. (**A**) PPI network showing the interactions between genes in the pathway. (**B**) Results of the KDA identifying key regulatory genes. Red nodes represent KDA-identified key driver genes, blue nodes denote seed genes, and gray nodes indicate associated genes.

Analysis of the ECM–receptor interaction pathway protein–protein interaction (PPI) network (Figure 4A) revealed multiple highly interconnected hub genes that may play central roles in ECM signaling and regulation. Notably, SMAD2, SMAD3, and SMAD4 emerged as top network hubs, all of which are known mediators of ECM–receptor signaling. The PPI network analysis not only demonstrated complex functional interactions among these genes but also provided a foundation for subsequent identification of key regulatory targets.

Key Driver Analysis (KDA) identified and ranked the top regulatory genes based on KDA scores (Figure 4B), revealing 10 critical driver genes within the ECM–receptor interaction pathway: ID1, GDF5, SMAD3, BMP4, TGFB2, BMPR2, ZFYVE16, ACVRL1, TGFB3, and DCN. Expression analysis (Table 1) demonstrated significant upregulation of ID1 and GDF5, along with marked downregulation of BMP4 and TGFB2, suggesting these genes may represent core molecular targets through which HSYA modulates ECM–receptor signaling dynamics.

### 2.5. SA-β-Gal Analysis

**Figure 5:**
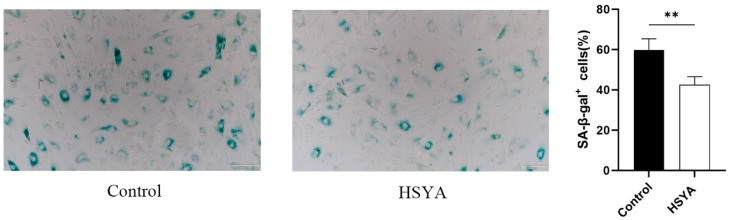
Effects of HSYA on SA-β-galactosidase activity in senescent cells. hUC-MSCs were stained for SA-β-gal and observed under an optical microscope (scale bar: 200 μm). Corresponding quantitation of cellular senescence ratios. Data are shown as mean ± SEM (*n* = 3). ** *p* < 0.01.

To evaluate the degree of cellular senescence, an SA-β-gal staining assay was conducted on passage 10 (P10) senescent hUC-MSCs that had undergone two rounds of passaging following HSYA treatment. As shown in Figure 5, HSYA treatment significantly reduced SA-β-gal activity and resulted in a noticeable reduction in staining intensity compared to the untreated group. These results suggest that HSYA exerts a moderating effect on the senescence process in hUC-MSCs.

### 2.6. Cell Cycle and Senescence-Related Protein Expression Analysis

**Figure 6:**
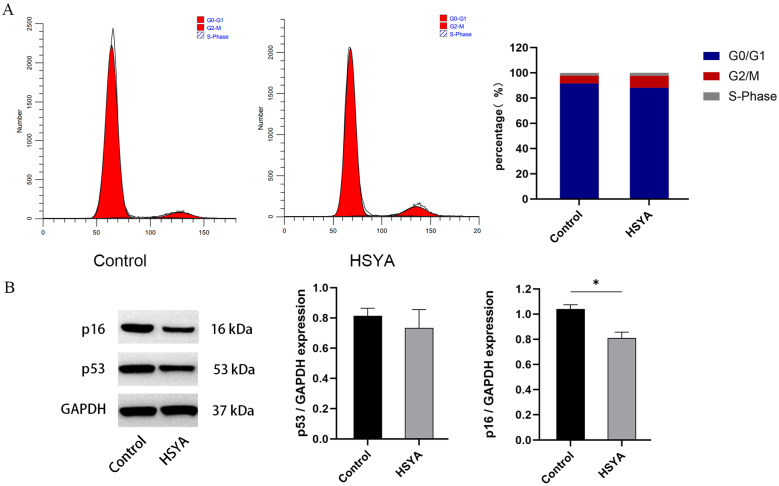
Effects of HSYA on cell cycle progression and senescence-related protein expression in senescent hUC-MSCs. (**A**) Cell cycle distribution analyzed by flow cytometry (PI staining). (**B**) Protein expression levels of p53 and p16 were assessed by Western blotting, with GAPDH serving as the internal control. Data are shown as mean ± SEM (*n* = 3). * *p* < 0.05.

Quantitative cell cycle analysis by flow cytometry (Figure 6A) demonstrated comparable distribution profiles between control and HSYA groups, with no statistically significant differences observed across all phases: G0/G1 (91.56% ± 1.2% vs. 88.14% ± 1.4%), G2/M (6.14% ± 2.3% vs. 9.54% ± 2.8%), and S phase (2.29% ± 0.8% vs. 2.31% ± 0.7%). These data suggest that HSYA treatment does not significantly modulate cell cycle progression under the tested experimental conditions.

Western blot analysis (Figure 6B) further indicated that HSYA treatment reduced the protein expression level of p16, whereas its effect on p53 levels did not reach statistical significance. Although HSYA effectively downregulated the expression of p16, a critical senescence-associated marker, it did not appear to induce substantial alterations in cell cycle distribution or p53 expression under the present experimental conditions.

This dissociation between p16 downregulation and stable p53 expression provides crucial protein-level evidence that HSYA ameliorates senescence potentially through a p53-independent pathway.

### 2.7. ROS Levels Analysis

**Figure 7:**
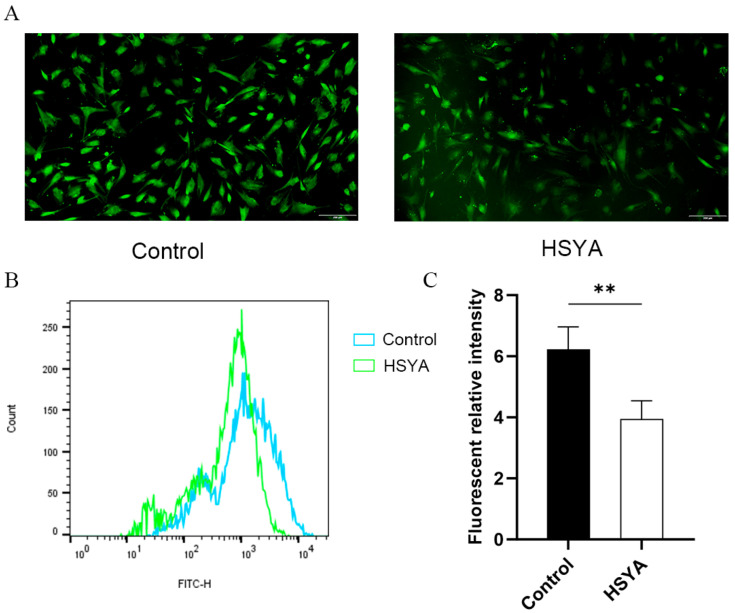
Effects of HSYA on intracellular ROS levels in senescent hUC-MSCs. (**A**) Representative fluorescent micrographs of control and 0.01 mg/mL HSYA-treated senescent hUC-MSCs after 72 h, stained with DCFH-DA to detect intracellular ROS. Scale bar, 200 μm. (**B**) Flow cytometry histograms showing DCF fluorescence intensity. (**C**) Quantitative analysis of relative fluorescence intensity normalized to the untreated control group. Data are shown as mean ± SEM (*n* = 3). ** *p* < 0.01.

To evaluate the effect of HSYA on oxidative stress levels in senescent hUC-MSCs, intracellular reactive oxygen species (ROS) levels were measured using the fluorescent probe DCFH-DA coupled with flow cytometry. Based on previous studies demonstrating the potent antioxidant activity of HSYA [26,27], we hypothesized that it could alleviate oxidative stress in senescent cells. As shown in Figure 7, compared with untreated senescent cells, HSYA treatment significantly reduced intracellular ROS levels. These results indicate that HSYA effectively attenuates excessive ROS accumulation in senescent hUC-MSCs, thereby ameliorating their oxidative stress status.

### 2.8. Gene Expression

**Figure 8:**
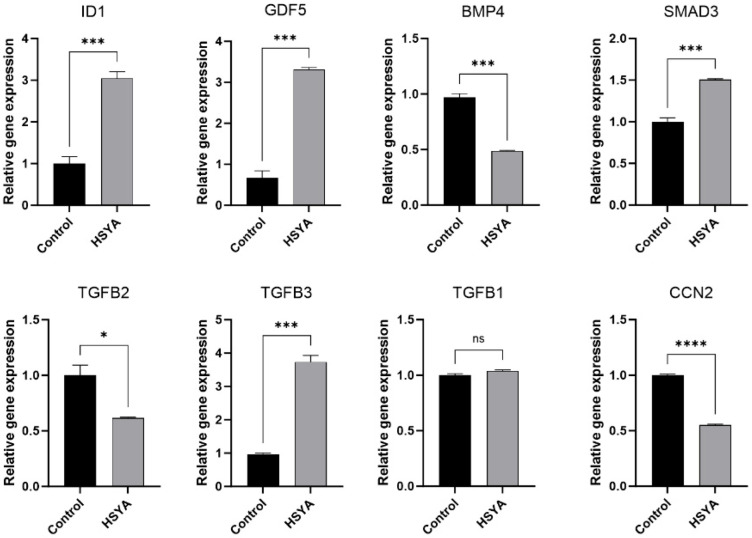
RT-qPCR validation of key drivers in the ECM–receptor interaction pathway. Control group is P10-MSCs. Data are shown as mean ± SEM (*n* = 3). Compared with control group, **** *p* < 0.0001, *** *p* < 0.001, * *p* < 0.05; ns, not significant.

The expression levels of genes (ID1, GDF5, BMP4, SAMD3, TGFB2, TGFB3, TGFB1 and CCN2) were measured by real-time quantitative PCR. As shown in Figure 8, HSYA treatment significantly upregulated the expression of ID1, GDF5, SMAD3, and TGFB3, while downregulating BMP4, TGFB2, and CCN2. No significant change was observed in TGFB1 gene expression.

## 3. Discussion

In this study, we first performed RNA-seq analysis to identify DEGs in HSYA-treated senescent hUC-MSCs. Among the significantly upregulated and downregulated genes, CCN2 and TGFB1 were notably downregulated. CCN2 is not only a pro-fibrotic effector molecule downstream of TGF-β but also independently enhances TGF-β signaling by upregulating TGF-β receptor II (TβRII) and activating the EGFR-SMAD pathway, thereby forming a positive feedback loop that drives fibrotic progression [28,29]. CCN2 establishes a dynamic interaction network with TGF-β and other cytokines via its characteristic multi-modular structure, creating a molecular platform for context-dependent signaling modulation. It not only synergistically enhances TGF-β signaling but also interferes with the pathway via ligand sequestration and receptor modulation. This context-dependent bidirectional regulatory property enables CCN2 to play a complex role in tissue homeostasis and disease progression [30]. HSYA treatment significantly down-regulated CCN2 expression in senescent hUC-MSCs, accompanied by reduced ROS levels. These results suggest that HSYA may ameliorate the fibrotic microenvironment of senescent mesenchymal stem cells by modulating the TGF-β signaling pathway.

Senescent cells exhibit a distinct secretory phenotype, which modulates the composition and organization of the surrounding ECM, thus regulating their microenvironment. Research indicates that the ECM of senescent mesenchymal stem cells (MSCs) exhibits significant structural and functional dysregulation, where reduced collagen synthesis coupled with increased matrix metalloproteinase-1 (MMP1) activity collectively contributes to ECM degradation [31].

ECM serves not merely as a structural scaffold for tissues, but more importantly as a dynamic biochemical signaling hub that regulates cellular behavior. Through the senescence-associated secretory phenotype (SASP), senescent cells actively remodel the ECM by disrupting collagen network architecture and promoting abnormal deposition of fibrotic proteins. These ECM modifications subsequently trigger activation of key signaling pathways, particularly the TGF-β/SMAD cascade, thereby creating a self-reinforcing pathological cycle where cellular senescence induces ECM disorganization, which in turn drives paracrine senescence propagation [32,33]. GO and KEGG enrichment analyses demonstrated the critical involvement of ECM–receptor interaction in HSYA-mediated functional modulation of senescent hUC-MSCs. HSYA alleviates tert-butyl hydroperoxide (TBHP)-induced oxidative stress damage and apoptosis in nucleus pulposus (NP) cell lines while improving the regulation of ECM homeostasis [34]. Furthermore, HSYA inhibits TGFB1-mediated Smad signaling by suppressing Smad2/3 phosphorylation and nuclear translocation, thereby blocking Smad3 binding to the type I collagen promoter [35]. Concurrently, studies demonstrate that HSYA improves vascular remodeling by suppressing TGF-β1, MMP-1, α-SMA and NF-κB p65 expression in adventitial fibroblasts, thereby reducing their proliferation and collagen synthesis [36]. These findings suggest that HSYA may alleviate excessive ECM deposition in senescent cells and promote ECM homeostasis in senescent hUC-MSCs by modulating the ECM–receptor interaction pathway.

Through KDA of the ECM–receptor interaction pathway, we identified a core regulatory module comprising ID1, GDF5 and SMAD3 that mechanistically contributes to HSYA-mediated attenuation of cellular senescence in hUC-MSCs. Notably, ID1 emerges as a central regulatory node exhibiting pleiotropic biological activities across multiple signaling tiers. ID1 functions as an effector molecule in the p53-dependent DNA damage response pathway. It promotes cell proliferation and counteracts senescence by inhibiting p21, while p53 negatively regulates ID1 expression through DEC1 [37]. Within the p53-NSC regulatory axis, ID1 emerges as a bi-directional mediator that converts p53 deficiency into both proliferation and differentiation outcomes through its dosage-sensitive control of stem cell fate decisions [38]. Additionally, in fibrotic skin disorders, ID1-b overexpression can inhibit TGF-β signaling by suppressing Smad2/3 phosphorylation, thereby negatively regulating TGF-β-driven collagen deposition [39]. GDF5 binds to specific receptors (including BMPR1b, BMPR2, and ACTR2a), activating Smad1/5/8 phosphorylation and promoting their nuclear translocation, which subsequently upregulates ID1 and ID3 expression [40]. Experimental validation shows that ID1 transcriptional activation by TGF-β1 in MCF10A cells is specifically mediated by SMAD3 [41]. Furthermore, while TGF-β-SMAD2/3 signaling drives ECM deposition and myofibroblast activation, BMP-SMAD1/5/8 exerts protective effects by competing for SMAD4 and inhibiting TGF-β receptors. The interplay between TGF-β and BMP pathways ultimately determines the progression of fibrosis [42].

Notably, although HSYA treatment significantly upregulated ID1 mRNA expression, it did not induce remarkable changes in p53 protein levels or alter cell cycle distribution. This suggests that HSYA may regulate ID1 through a p53-independent mechanism. Although ID1 can promote proliferation by inhibiting cyclin-dependent kinase inhibitors (CKIs) such as p21, the full manifestation of its effects may require additional synergistic signals or a specific cellular context [37]. Furthermore, p53 activity depends not only on its total protein abundance but also on post-translational modifications (e.g., phosphorylation, acetylation), subcellular localization (particularly nuclear translocation), and interactions with co-activators or repressors [43,44].

Although Western blot results indicated that HSYA did not alter total p53 protein levels, it may subtly modulate its post-translational modification status (such as phosphorylation or acetylation), thereby altering its functional activity and influencing the transcription of specific downstream target genes—without sufficient magnitude to elicit significant fluctuations in p53 abundance or drive global cell cycle re-entry. The concurrent upregulation of GDF5 and SMAD3, key components of the BMP signaling pathway, provides a plausible p53-independent mechanism for ID1 induction by HSYA.

HSYA significantly downregulated pro-fibrotic factors such as CCN2 and TGFB1 and modulated ECM–receptor interactions. These effects may primarily contribute to improved cellular function and amelioration of the fibrotic microenvironment. Collectively, this evidence indicates that under HSYA intervention, ID1 may function mainly as a modulator of microenvironmental homeostasis, contributing to enhanced cellular function through coordination of ECM remodeling and TGF-β/BMP signaling balance, rather than directly reversing cell cycle arrest.

The composition and physical properties of the ECM influence cell cycle progression through integrin-mediated adhesion complexes [45]. However, flow cytometry analysis in our study revealed no significant alterations in cell cycle phase distribution following HSYA treatment. Western blot analysis in this study confirmed that HSYA significantly downregulated p16 protein expression. However, the cell cycle arrest established by the p16/Rb pathway in replicatively senescent hUC-MSCs is stringent and frequently permanent. Based on these experimental findings, HSYA may reduce p16 protein levels without markedly altering p53 expression, thereby inducing only a partial reversal of Rb-mediated G1/G0 phase arrest [46,47,48]. This suggests that HSYA does not directly drive cell cycle progression. Furthermore, the senescent cell population likely exhibits substantial heterogeneity [49], wherein HSYA may only partially attenuate functionality in early-stage senescent cells while remaining ineffective against the epigenetic blockade in deeply senescent populations.

In summary, our study demonstrates that HSYA treatment induces a distinct differential expression of key molecules within the TGF-β and BMP signaling pathways. This specific regulatory pattern suggests that HSYA ameliorates senescent phenotypes in hUC-MSCs primarily by modulating the ECM–receptor interaction pathway and remodeling the TGF-β/BMP signaling crosstalk network, rather than directly reversing cell cycle arrest. However, we acknowledge the limitations of our current work. While we propose a p53-independent mechanism for ID1 induction, the potential modulation of p53 activity via undetected post-translational modifications cannot be ruled out. Furthermore, the precise functional role of ID1 in coordinating the TGF-β/BMP balance remains to be fully validated. Therefore, future studies should not only incorporate the dynamic immunomodulatory properties of MSCs, cellular heterogeneity, and microenvironmental interactions but also employ direct functional experiments, such as ID1 gain- and loss-of-function studies, to definitively test this model and optimize anti-aging strategies.

## 4. Materials and Methods

### 4.1. Cell Culture

hUC-MSCs were acquired from our laboratory (Key Laboratory of School of Life Sciences, Beijing University of Chinese Medicine, Beijing, China). A replicative senescence model was established through serial passaging, with cells at passage 10 designated as senescent. Prior to cell culture, the culture flasks were coated with NutriCoat™ Attachment Solution (Sartorius, Göttingen, Germany). The cells were cultivated in MSC NutriStem^®^XF Basal Medium, which was obtained from Sartorius in Germany. To support cell growth, the medium was supplemented with MSC NutriStem^®^ XF Supplement Mix also from Sartorius, along with 1% Penicillin-Streptomycin Solution. The cells were maintained at a temperature of 37 °C, with a CO_2_ concentration of 5%.

### 4.2. DEG Analysis

To establish a replicative senescence model, P10 presenescent hUC-MSCs were designated as the senescence group, while parallel P10 cultures treated with 0.01 mg/mL HSYA comprised the HSYA group (1 × 10^5^ cells/well in 6-well plates). Total RNA was extracted from three independent biological replicates per group using a kit (TIANGEN, Beijing, China), followed by quality assessment via UV-spectrophotometry. Libraries constructed from enriched mRNA were sequenced on the BGI Genomics platform. Raw data were processed with SOAPnuke (v1.5.0), Bowtie2 (v2.2.5), and Samtools (v1.2) to generate Clean Data. Differential expression analysis (|log_2_FC| > 1.5, *p* < 0.05) was performed using DESeq2 via Dr.TOM (biosys.bgi.com), accessed on 15 July 2025, with results visualized in volcano plots.

### 4.3. GO Functional and KEGG Pathway Enrichment Analysis

GO functional enrichment was performed using the Metascape platform with reference to the GO database. The hypergeometric test was applied to calculate the enrichment significance of each GO term, with significantly enriched terms identified at *p* < 0.05. DEGs were subjected to KEGG pathway enrichment analysis. Significantly enriched pathways were identified following multiple testing correction (e.g., Benjamini-Hochberg) to control the false discovery rate.

### 4.4. GSEA and PPI Network Construction

The 2766 DEGs obtained from sequencing were subjected to KEGG annotation and GSEA) using the algorithm developed by the Broad Institute. This analysis identifies the most significantly altered gene sets in the selected samples, focusing on signaling pathways, biological processes, or functional components. The PPI network was constructed based on known interactions from the STRING11 database and transcript mapping relationships from NCBI Reference. In the STRING11 database, a higher Score indicates a more reliable PPI, with a default threshold of 500. Additionally, KDA was performed to identify critical regulatory genes that occupy central positions in the network, also based on the STRING11 database.

### 4.5. SA-β-Gal Measurement

Senescent cells were seeded into 12-well plates at a density of 5 × 10^4^ cells per well, with three replicate wells per group. The senescent cells were treated continuously with 0.01 mg/mL HSYA and expanded for two passages. According to the manufacturer’s instructions (Solarbio, Shanghai, China), cells were fixed with β-galactosidase fixation solution for 15 min and washed three times with PBS. SA-β-gal staining working solution was then prepared. Cells were incubated overnight at 37 °C in SA-β-gal staining solution (pH 6.0) without CO_2_. The number of positively stained cells was counted in six randomly selected fields under an optical microscope. The percentage of SA-β-gal-positive cells was calculated as follows: (number of positive cells/total number of cells in the same field) × 100%.

### 4.6. Cell Cycle Assay

hUC-MSCs were cultured in 6-well plates and treated with 0.01 mg/mL HSYA for 72 h. Cells were harvested using Tryple, fixed in 70% ethanol at 4 °C overnight, then stained with PI/RNase A solution (50 μg/mL PI, 100 μg/mL RNase A) at 37 °C for 30 min. After filtration through a 200-μm mesh, cell cycle distribution was analyzed by flow cytometry using ModFit software (version 5.0) to determine G0/G1, S, and G2/M phase percentages.

### 4.7. ROS Measurement

Measurement of intracellular ROS levels was performed using the fluorescent probe 2′,7′-dichlorodihydrofluorescein diacetate (DCFH-DA). Senescent and HSYA-treated cells were seeded in 6-well plates with three replicate wells per group. According to the manufacturer’s instructions of the ROS assay kit (Beyotime, Shanghai, China), the old culture medium was removed, and the cells were washed once with phosphate-buffered saline (PBS). DCFH-DA was diluted 1:1000 in serum-free medium to achieve a final concentration of 2 μmol/L and then added to the cells. The cells were incubated with the probe for 30 min at 37 °C in the dark. After incubation, the cells were washed twice with serum-free medium to remove any unbound probe. Intracellular ROS levels were subsequently detected and quantified using both fluorescence microscopy and flow cytometry.

### 4.8. RT-qPCR Analysis

Cells were seeded into 6-well plates. After 72 h of treatment with HSYA, senescent hUC-MSCs were harvested for quantitative real-time polymerase chain reaction (qRT-PCR) analysis to evaluate the expression levels of ID1, GDF5, BMP4, SMAD3, TGFB2, TGFB3, TGFB1, and CCN2. Total RNA was extracted using the RNA Easy Fast Cell Kit (TIANGEN, Beijing, China). The concentration and purity of the RNA were determined with a NanoDrop One spectrophotometer (Thermo Scientific, Waltham, MA, USA), and integrity was confirmed by agarose gel electrophoresis. First-strand cDNA was synthesized from 1 μg of total RNA using the First-strand cDNA Synthesis Mix (Lablead, Beijing, China). qRT-PCR was performed using a 20 μL reaction mixture containing 10 μL of Taq SYBR Green qPCR Premix (Lablead, Beijing, China), 10 ng of cDNA, and specific primers purchased from OriGene Technologies Inc. (Rockville, MD, USA). The primer sequences and annealing temperatures for each gene are listed in Table 2. The PCR protocol consisted of an initial denaturation at 95 °C for 30 s, followed by 40 cycles of 95 °C for 10 s and 60 °C for 30 s. Melting curve analysis was carried out at 95 °C for 15 s, 60 °C for 60 s, and 95 °C for 30 s. Gene expression was quantified using the comparative 2^−ΔΔCt^ method, with normalization to the expression of an appropriate housekeeping gene.

### 4.9. Western Blot Analysis

Total protein was extracted from cells of each group using RIPA lysis buffer supplemented with protease inhibitors. The proteins were separated by 10% SDS-PAGE and subsequently transferred onto PVDF membranes. The membranes were blocked with 5% skim milk prepared in 1× TBST for 1.5 h at room temperature. Then, they were incubated with primary antibodies against p53 and p16 (Proteintech; diluted 1:1000) at 4 °C overnight with gentle shaking. After incubation, the membranes were washed three times with TBST and incubated with corresponding HRP-conjugated secondary antibodies (diluted 1:3000) for 1 h at room temperature with shaking. Following three additional washes with TBST, the blots were developed using an ECL working solution and imaged with a fully automated chemiluminescence imaging analysis system. Protein band intensities were quantified using GelPro32 software (version 4.0).

### 4.10. Data Analysis

The data were analyzed using GraphPad Prism (version 9.0). All experiments were performed in triplicate, and data are presented as mean ± SEM. Differences between two groups were assessed using unpaired Student’s *t*-tests. *p*-value < 0.05 was considered statistically significant.

## Figures and Tables

**Table 1:** Top 10 Key Driver Genes Identified by KDA.

Gene Symbol	Control Average FPKM	HSYA Average FPKM	log_2_ (HSYA/Control)	Q-Value
ID1	4.35	144.62	4.79	1.71 × 10^−88^
GDF5	0.29	12.31	5.21	6.58 × 10^−46^
SMAD3	3.21	13.31	1.89	4.33 × 10^−37^
BMP4	14.04	3.01	−2.37	2.24 × 10^−25^
TGFB2	19.54	2.86	−3.03	4.52 × 10^−17^
ZFYVE16	6.75	1.95	−1.91	1.40 × 10^−13^
BMPR2	18.71	4.75	−2.22	5.02 × 10^−13^
ACVRL1	0.71	4.34	2.47	6.16 × 10^−13^
TGFB3	0.21	1.77	2.87	9.66 × 10^−13^
DCN	129.94	39.06	−1.95	1.31 × 10^−12^

**Table 2:** Primer sequences for PCR amplification.

Gene Symbol	Forward Primer (5′-3′)	Reverse Primer (5′-3′)
ID1	GTTGGAGCTGAACTCGGAATCC	ACACAAGATGCGATCGTCCGCA
GDF5	AACAGCAGCGTGAAGTTGGAGG	ACACGTACCTCTGCTTCCTGAC
SMAD3	TGAGGCTGTCTACCAGTTGACC	GTGAGGACCTTGTCAAGCCACT
BMP4	CTGGTCTTGAGTATCCTGAGCG	TCACCTCGTTCTCAGGGATGCT
TGFB2	AAGAAGCGTGCTTTGGATGCGG	ATGCTCCAGCACAGAAGTTGGC
TGFB3	CTAAGCGGAATGAGCAGAGGATC	TCTCAACAGCCACTCACGCACA
TGFB1	TACCTGAACCCGTGTTGCTCTC	GTTGCTGAGGTATCGCCAGGAA
CCN2	CTTGCGAAGCTGACCTGGAAGA	CCGTCGGTACATACTCCACAGA
β-actin	GGGAAATCGTGCGTGACATT	AGGTAGTTTCGTGGATGCCA

## Data Availability

Dataset available on request from the authors.
